# Glucocorticoid-dependent expression of IAP participates in the protection against TNF-mediated cytotoxicity in MCF7 cells

**DOI:** 10.1186/s12885-019-5563-y

**Published:** 2019-04-15

**Authors:** Irma B. Mitre-Aguilar, Tonatiuh Barrios-Garcia, Victor M. Ruiz-Lopez, Alberto J. Cabrera-Quintero, Nancy R. Mejia-Dominguez, Jose L. Ventura-Gallegos, Daniel Moreno-Mitre, Alejandro Aranda-Gutierrez, Janini Mejia-Rangel, Alma R. Escalona-Guzman, Yanin Chavarri-Guerra, Alfonso Leon-Del-Rio, Alejandro Zentella-Dehesa

**Affiliations:** 1 Departamento de Medicina Genomica y Toxicologia Ambiental, Instituto de Investigaciones Biomedicas (IIBO), Universidad Nacional Autonoma de Mexico (UNAM), 04510 Ciudad de Mexico (CDMX), Mexico, Mexico; 2 Programa de Investigacion en Cancer de Mama, IIBO, UNAM, 04510 Mexico, CDMX Mexico; 3 Unidad de Bioquimica, Instituto Nacional de Ciencias Medicas y Nutricion Salvador Zubiran (INCMNSZ), 14080 Mexico, CDMX Mexico; 4 Centro de Cancer, Centro Medico ABC, 01120 Mexico, CDMX Mexico; 5 Departamento de Biologia Molecular y Biotecnologia, IIBO, UNAM, 04510 Mexico, CDMX Mexico; 6 Departamento de Biologia Molecular, Instituto Nacional de Enfermedades Respiratorias (INER), 14080 Mexico, CDMX Mexico; 7 Red de Apoyo a la Investigacion-Coordinacion de la Investigacion Cientifica (RAI-CIC), UNAM, 14080 Mexico, CDMX Mexico; 8 Departamento de Hemato-Oncologia, Instituto Nacional de Ciencias Medicas y Nutricion Salvador Zubiran, 14080 Mexico, CDMX Mexico

**Keywords:** Cell death, Glucocorticoid receptor, Glucocorticoids, Inhibitor of apoptosis proteins, Tumor necrosis factor, MCF7 cells

## Abstract

**Background:**

Glucocorticoid receptor (GR) activation has been associated with breast cancer cell survival in vitro. Glucocorticoid (GC)-dependent protection against tumor necrosis factor (TNF)-induced cell death has been well characterized in MCF7 luminal A breast cancer cells. The GR activates a variety of protective mechanisms, such as inhibitors of apoptosis proteins (IAPs). However, the relative contribution of the GR-dependent expression of IAPs in the protection of cell death has not, to our knowledge, been evaluated.

**Methods:**

MCF7 cells were used for all experiments. GR was activated with cortisol (CORT) or dexamethasone (DEX) and inhibited with mifepristone (RU486). Cell viability was determined in real-time with the xCELLigence™ RTCA System and at specific endpoints using crystal violet stain. The mRNA levels of the eight members of the IAP family were measured by qRT-PCR. The protein levels of GR, PR, ERα, HER2, PARP1, c-IAP1 and XIAP were evaluated by Western blot analysis. The knockdown of c-IAP1 and XIAP was accomplished via transient transfection with specific siRNAs. GR activation was verified by a gene reporter assay. Via the cBioportal interphase we queried the mRNA levels of GR and IAPs in breast cancer tumors.

**Results:**

RU486 significantly inhibited the anti-cytotoxic effect of both GCs. PARP1 processing was diminished in the presence of both GCs. The combined treatments of GCs + TNF increased the relative mRNA levels of Survivin>c-IAP1 > NAIP>Apollon>XIAP>Ts-IAP > ML-IAP > c-IAP2. Additionally, GR mRNA content increased with the combined treatments of GCs + TNF. Sustained levels of the proteins c-IAP1 and XIAP were observed after 48 h of the combined treatments with GCs + TNF. With *c-IAP1* and *XIAP* gene silencing, the GC-mediated protection was diminished. In the breast tumor samples, the GR mRNA was coexpressed with Apollon and XIAP with a Pearson coefficient greater than 0.3.

**Conclusions:**

The effect of GCs against TNF-mediated cytotoxicity involves increased mRNA expression and sustained protein levels of c-IAP1 and XIAP. The antagonist effects of RU486 and the qRT-PCR results also suggest the role of the GR in this process. This finding may have clinical implications because the GR and IAPs are expressed in breast tumor samples.

**Electronic supplementary material:**

The online version of this article (10.1186/s12885-019-5563-y) contains supplementary material, which is available to authorized users.

## Background

The expression of hormone receptors (ERα receptor and PRβ receptor) and the HER2 receptor are key biomarkers for breast cancer subtyping, prognosis, and therapy [1]. However, breast cancer cells express additional families of hormone receptors, especially the nuclear receptor superfamily [2]. The nuclear expression of glucocorticoid (GC) receptors (GRs) in breast cancer samples of all intrinsic subtypes has been demonstrated by immunohistochemistry (IHC). However, GR expression levels might vary according to estrogen and progesterone expression and tumor grade. The prognostic value of GR expression over other clinical features has not been proven [3]. Breast cancer patients are continuously exposed to GR agonists. For example, cortisol (CORT) is present in the blood of patients with breast cancer regardless of age; this represents a sharp contrast to estrogen levels [4]. It has been reported that the deregulation of circadian rhythms may increase CORT exposure time in breast cancer patients [5]. Additionally, to manage the secondary effects of chemotherapy, breast cancer patients are administered synthetic GR agonists, especially dexamethasone (DEX) [6]. GR agonists, such as CORT and DEX, exert pleiotropic effects on the breast tumor microenvironment [7]. Due to the relevance of CORT and DEX in cancer, their effects on cancer cell proliferation and death have been extensively studied [8]. However, the results of these studies are an intense area of debate. Some studies point out that GCs hinder proliferation [9], while others indicate that GCs induce proliferation [10]. However, current evidence suggests that GCs consistently counteract the effect of cytotoxic agents, reducing cell death [11].The mechanism by which GCs confer protection against cell death has not been fully elucidated. However, the use of small interfering RNA (siRNA) to inhibit the expression of the kinase SGK1 [12] and the phosphatase dual specificity phosphatase 1 (DUSP1) [13] revealed that these two enzymes are necessary for the GC-mediated protective effect. Moreover, GC treatment induced the downregulation of the nuclear factor-kappa beta (NF-κB)-dependent expression of first apoptosis signal receptor (FAS) [10]. The current understanding of the GC-induced interference with cell death is well documented in experiments where tumor necrosis factor (TNF) is used to cause cell death [14]. Previously, it was established that MCF7 cells express cell inhibitor of apoptosis proteins (IAPs), such as cell-IAP-1 (c-IAP1) and X chromosome-linked IAP (XIAP) [15]. TNF treatment decreases the expression of these IAPs, and DEX prevents this decrease in an NF-κB-dependent manner [16]. Therefore, we tested the protective effect of CORT as well as DEX, and we evaluated the relative participation of c-IAP1 and XIAP in the interference of GC-mediated cell death with transient RNAi.

## Methods

### Materials

CORT, DEX, and RU486 were obtained from Sigma-Aldrich (St. Louis, MO, USA), and human recombinant TNF was obtained from R&D Systems, Inc. (Minneapolis, MN, USA). Cell culture media and sera were obtained from Invitrogen Life Technologies (San Diego, CA, USA). Rabbit polyclonal IgG antibodies against c-IAP1 and XIAP were obtained from Upstate (Merck, Darmstadt, Germany). Protease inhibitor cocktail tablets were obtained from Boehringer Mannheim (East Sussex, UK). Secondary antibodies were purchased from Pierce Biotechnology, Inc. (Rockford, IL, USA) (anti-rabbit IgG) and Zymed Laboratories (Carlsbad, CA, USA) (anti-mouse and anti-goat IgG). The SuperSignal™ West Pico PLUS Chemiluminescent Substrate was purchased from ThermoFisher Scientific (Waltham, MA, USA). The *Escherichia coli* DH5α strain was obtained from Gibco BRL (Paisley, UK) and was subcloned into the expression vector pcDNA3.1-GR under the control of the cytomegalovirus pCMV-βGal promoter.

### Plasmids

The pcDNA3.1-GR and GRE-Tk-LUC vectors were kindly provided by Dr. W. Lee Kraus (Cornell University), amplified by RT-PCR and cloned into the mammalian expression vector pcDNA3.1 from Invitrogen (Carlsbad, CA, USA).

### Cell culture

The luminal A breast cancer cell line MCF7 (ATCC® HTB¬22™) containing nuclear GR (see Additional file 4) was obtained from the American Type Culture Collection (ATCC, Manassas, VA, USA) and maintained in minimum Eagle’s medium supplemented with 10% (v/v) inactivated fetal bovine serum (FBS) (GIBCO, Rockville MD, USA), 100 U/mL penicillin, 100 μg/mL streptomycin, and 0.25 μg/mL amphotericin B (GIBCO, Rockville MD, USA) in a humidified atmosphere containing 5% CO_**2**_ at 37 °C.

### Cell death assay

The MCF7 (1.5 × 10^4^/cm^2^) cell line was stimulated with a final concentration of 10 μM of CORT, DEX, or RU486 and/or 10 ng/mL of human recombinant TNF. Cell viability was measured by a crystal violet staining assay in a 48-well plate, and cells were fixed at 24, 48, and 72 h after cell treatment, with the addition of 200 μL of 1.1% glutaraldehyde at the end of each experiment. Afterwards, the plates were stained with 500 μL of crystal violet staining solution (0.1% crystal violet in 10% formic acid) for 20 min. The excess crystal violet staining solution was removed with distilled water, and the cells were air-dried. The crystal violet stain bound to the samples was dissolved with 500 μL of 10% acetic acid. Then, 150 μL of the solution was placed into 96-well plates and quantified at 590 nm in an ELISA plate reader.

### xCELLigence™ viability assay

Dynamic monitoring of MCF7 cell viability was performed with the xCELLigence™ RTCA System. (ACEA Biosciences, San Diego CA, USA). MCF7 cells were seeded (1.5 × 10^4^ cells/cm^2^) on an E-plate-16 at the optimal cell density for the cell proliferation assay. Cell growth curves were recorded on the xCELLigence™ RTCA System in real-time every 30 min. Cells adhered to the bottom of each well, covering the surface of the sensor that monitors cells by measuring their normalized cell index (NCI). The NCI was dynamically recorded in real-time without labeling the cells. The RTCA DP instrument utilizes the E-plate-16 for the cell death assay. Impedance is correlated with an increase in the number of cells that are on the underside of the well by measuring NCI.

### Gene reporter assay

MCF7 cells (2 × 10^5^) were seeded into six-well tissue culture dishes containing phenol red-free RPMI supplemented with 10% charcoal/dextran-treated FBS (stripped FBS) and cultured for 24 h. Then, the cells were transfected by employing the calcium phosphate-DNA [Ca_3_(PO4)_2_] coprecipitation method, which typically included 2 g of GRE-Tk-LUC reporter, 0.1 g of pCMV-βGal (transfection control), and 0.25–1.0 μg pcDNA3.1-GR or another test vector. After 6 h, the cells were washed twice with a phosphate-buffered saline (PBS) solution and treated with either 10 μM of CORT, 10 μM of DEX, 10 ng/mL of TNF, or carrier (0.01% ethanol) for 24 h in phenol red-free RPMI supplemented with 5% stripped FBS. The cells were then washed and harvested in a potassium phosphate lysis buffer containing 1% Triton X-100. Luciferase and β-galactosidase activities were measured using a Monolight 3010 Luminometer (Pharmingen).

### siRNA assay

To suppress c-IAP1 and XIAP expression, the following targeted siRNA pools were administered according to the manufacturer’s instructions: human-siRNA GS331 XIAP (#1027416); human-siRNA GS329 c-IAP1 (#1027416); silencer negative control-siRNA (#1027280); and cell death control-siRNA (#1027298). The siRNAs and the transfection reagent (#301705) were obtained from QIAGEN Biotechnology (Cambridge, MA, USA). The reduction in c-IAP1 and XIAP expression was determined by Western blot (WB) analysis employing anti-c-IAP1 and anti-XIAP antibodies.

### Western blot analysis

MCF7 cells (1.5 × 10^4^ cells/cm^2^) were cultured in 100 mm cell culture dishes for 24 h after stimulation with 10 μM of CORT, 10 μM of DEX, and/or 10 ng/mL of TNF. The cells were lysed at predetermined intervals after treatment: 1, 3, 6, 12, 24, and 48 h. To detect nuclear receptors, we used BT-474 cells (ATCC® HTB-20™, ductal carcinoma HER2/neu-positive cells). Cell extracts were lysed in RIPA buffer (50 mM of Tris, 5 mM of EDTA, 150 mM of NaCl, 0.5% of Nonidet-40, 1 mM of PMSF, pH 8.0) followed by centrifugation (4000 *g* for 5 min). The protein concentrations in total cell lysates were measured using the Bradford assay with bovine serum albumin (BSA) as the standard curve. Whole cell lysates (30 μg) were eluted with 5X Laemmli buffer, electrophoresed in 10% Tris-glycine polyacrylamide gels, and separated by SDS-PAGE. WB analysis was performed using the following specific primary rabbit polyclonal IgG antibodies: anti-c-IAP1 (1:1500 dilution; Upstate catalog #07–759; Merck, Darmstadt, Germany); anti-XIAP (1:2000 dilution; Upstate catalog #07–753; Merck Darmstadt, Germany); anti-mature PARP1 (1:200 dilution; R&D Systems, Minneapolis, MN, USA); anti-cleaved PARP1 (1:200 dilution; R&D Systems, Minneapolis, MN, USA); and anti-HER2/neu receptor (1:3000 dilution; Santa Cruz Biotechnology SC-284; Dallas, Texas, USA). The following additional mouse IgG primary antibodies were used in the experiments: anti-GRα receptor (1:500 dilution; Santa Cruz Biotechnology SC-393232); anti-PRβ receptor (1:100 dilution; Santa Cruz Biotechnology SC-810); anti-ERα receptor (1:250 dilution; Santa Cruz Biotechnology SC-8005); and anti-β-actin (1:10,000 dilution; R&D Systems, Minneapolis, MN, USA). The anti-rabbit (1:10,000 dilution), IgG (1:10,000 dilution), and anti-goat (1:5,000 dilution) secondary HRP-conjugated antibodies were purchased from Zymed (San Francisco, CA), and the anti-mouse IgG (1:15,000 dilution) was obtained from Pierce (Rockford, IL, USA). The proteins were visualized on X-ray film using an enhanced chemiluminescence assay using the SuperSignal™ West Pico PLUS Chemiluminescent Substrate from ThermoFisher Scientific (Waltham, MA, USA).

### qRT-PCR analysis

One microgram of total RNA was treated with 1 unit of DNAse for 30 min at 37 °C. Complementary DNA (cDNA) was synthesized using a High-Capacity cDNA Reverse Transcription Kit from Applied-Biosystems (Foster CA, USA) according to the manufacturer’s specifications. Briefly, a master mix was prepared using 50 U of multiscribe retro-transcriptase (RT) enzyme, 0.8 μL of dNTP mix (100 mM total), 2.0 μL of RT buffer 10X, 3.8 U of RNase inhibitor, 1 μL of random primers (10X), and 1 μg of total sample RNA in a final volume of 15 μL completed with free nuclease water. The mix was incubated at 25 °C for 10 min, followed by incubation at 37 °C for 1 h and at 85 °C for 5 s. cDNA was used to quantify transcript abundance through real-time PCR (qRT-PCR) using specific primers (Additional file 1) in a Step-One-System from Applied-Biosystems (Foster CA, USA). The amplification reaction contained 200 ng of cDNA, 7.5 μL of a master mix (SYBR Green) from Applied-Biosystems (Foster CA, USA), 10 pmol of each primer in a final volume of 15 μl, completed with nuclease-free water. PCR cycling conditions were 95 °C for 2 min, followed by 40 cycles of 95 °C for 15 s and 60 °C for 1 min. After 40 cycles, the Delta-CT method was performed to compare the levels of the transcripts. The results were expressed as 2 elevated to the negative power of the difference between CT-TNF and CT-IAP minus CT-ethanol (ETOH) (2-^Δct^). The specificity of PCR was determined with the melt curve program.

### Bioinformatic analysis

The data were retrieved from a public online resource [17, 18]. Using this resource, the public and curated database The Breast Invasive Carcinoma (TCGA, provisional) was accessed. mRNA expression z-Scores (RNA-seq V2 RSEM) for *NR3C1* and for the eight members of the *IAP* gene family were selected. Because the classification of tumor samples into the luminal A, luminal B, HER2+, and triple negative breast cancer (TNBC) categories was not available, IHC data describing ERα, HER2, and PRβ status were downloaded. The tumor samples were separated into the correct intrinsic subtype category based on their positive or negative status of ERα, PRβ, and HER2 as determined by IHC. The scatter plot of *NR3C1* mRNA vs intrinsic subtype was created using Microsoft Excel software. The graphs and table comparing *NR3C1* with members of the *IAP* gene family were directly retrieved from the plot tab of cBioportal.

### Statistical analysis

Each experiment was performed at least three times. Data are presented as the mean ± standard deviation (SD). Data were analyzed using one-way analysis of variance (ANOVA) followed by the Bonferroni method for multiple comparisons. A level of *p < 0.05 was considered statistically significant.

For two siRNA experiments, the effect of the treatment on cell viability for each siRNA was examined with ANOVA. In the case of c-IAP1 and XIAP, we employed a general linear model (GLM) with gamma error distribution. Additionally, we examined the interaction of siRNA type and treatment through deviance analysis. Tukey post hoc analysis was performed in the respective comparisons.

## Results

### Cortisol and dexamethasone impair the TNF-induced death of MCF7 cells

**Fig. 1:**
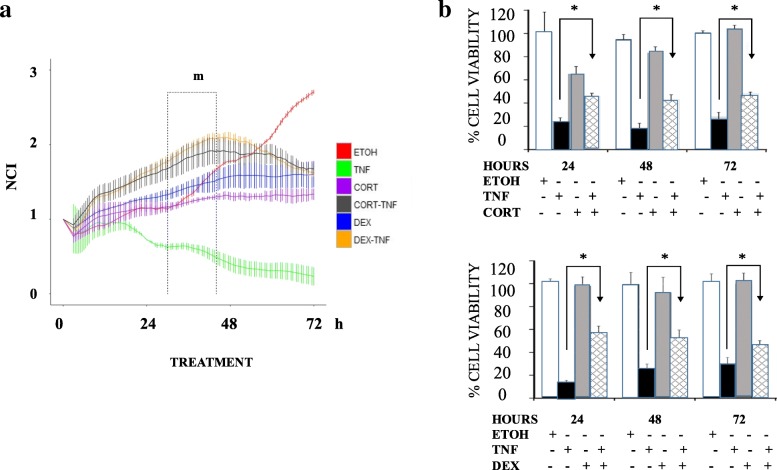
Dexamethasone and cortisol block the cytotoxic effect of TNF on MCF7 cells. **a** Normalized cell index (NCI) and curves of MCF7 cells treated for 72 h (left panel). ETOH = red, TNF = green, CORT = purple, CORT + TNF = black, DEX = blue, and DEX + TNF = orange. Comparisons of the NCI at 24, 48, and 72 h. Vertical lines represent the standard deviation (SD) of each measured point (Right panel). **b** Viability courses of MCF7 cells treated for 24, 48, and 72 h. Top panel: ETOH, TNF, CORT, and CORT + TNF. Bottom panel: Treatment with DEX instead of CORT (*p < 0.05)

Significant reductions in the total number of cells and the number of cells with a spiculated morphology were observed in the 48 h TNF treatment compared to control cells that received only vehicle (ETOH) treatment. None of these changes were present when TNF was added with CORT or DEX. The addition of GCs resulted in a cytostatic effect; cell density decreased by 76 and 67% with CORT and DEX, respectively, while the occurrence of mitotic figures decreased by 35 and 29% with CORT and DEX, respectively. (Additional file 2). The NCI was measured in real-time every 30 min for 72 h with the xCELLigence™ system (Fig. 1a). MCF7 cells were treated as described above. We analyzed the rate of change and expressed this value as NCI/30 min. The results showed that the control cells (Fig. 1a, red line) increased in three stages: 1° from 3 to 21 h with slope = + 0.0186; 2° from 30 to 44 h with slope = + 0.0375; and 3° from 52 to 72 h with slope = + 0.0432. TNF treatment (Fig. 1a, green line) decreased in three stages: 1° from 14 to 27 h with slope = − 0.0233; 2° from 40 to 50 h with slope = − 0.0208; and 3° from 50 to 72 h with slope = − 0.0062. The combinations of CORT + TNF (Fig. 1a, black line) or DEX + TNF (Fig. 1a, orange line) exhibited two increasing stages followed by one decreasing stage: 1° from 3 to 12 h with slope = + 0.0566; 2° from 10 to 42 h with slope = + 0.0187; and decreasing from 62 to 72 h with slope = − 0.0161. We compared the proliferation rates of the six experimental groups (Fig. 1a, time window m) from 30 to 44 h. The MCF7 control cells grew at a rate of 0.025 NCI/h (100%); however, with the GCs treatments, growth decreased to one-half of the control growth (0.009 (38%) and 0.014 (55%) for CORT and DEX, respectively). With the combined GCs + TNF treatments, the proliferation rates improved (0.016 (65%) and 0.022 (87%) for CORT + TNF and DEX + TNF, respectively). In contrast, TNF treatment alone presented a strong negative growth rate (− 0.01 NCI/h), reflecting cell death (*p < 0.001; Fig. 1a and Additional file 3); TNF treatment resulted in the lowest NCI values with respect to the other treatments at 24, 48, and 72 h for the different treatments. In the CORT + TNF combination treatment group, the average NCI values were 2.3, 4.8, and 8 times those in the TNF group at 24, 48, and 72 h, respectively. In the DEX + TNF combination treatment group, the average NCI values were 2.3, 5.4, and 7.6 times those in the TNF group at 24, 48, and 72 h, respectively. The protective effect of both GCs was very similar over time: After 24 h, the NCI values of the CORT + TNF and DEX + TNF groups were 2.3 times that of the TNF group; by 48 h, these NCI values were close to 5 times that of the TNF group; and by 72 h, there was a 7-fold difference. These results suggest that the protective effects of the two GCs tested are very similar (Additional file 3).

Taken together, these results suggest that while TNF induced cell death, the addition of GCs prevented cell death and allowed for cell proliferation up to 45 h. GCs alone appear to exert a cytostatic effect after 48 h. The results demonstrated that both GCs result in similar anti-cytotoxic protection.

### The anti-cytotoxic protection conferred by cortisol or dexamethasone is mediated by the glucocorticoid receptor pathway

**Fig. 2:**
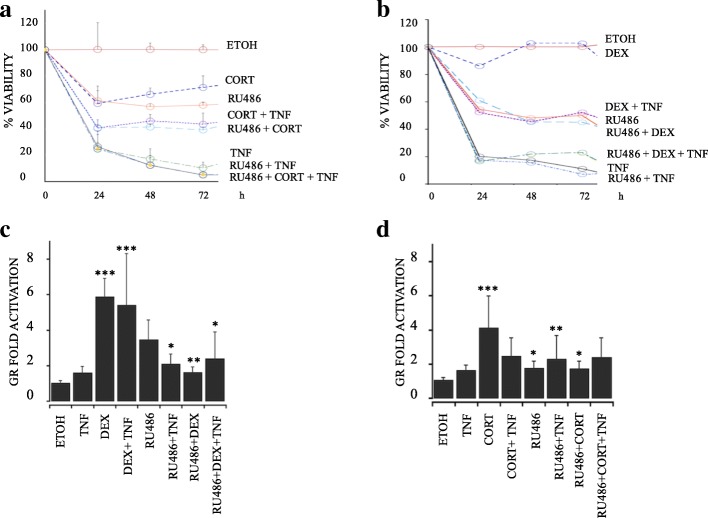
Protection against cytotoxicity provided by cortisol (CORT) and dexamethasone (DEX) is mediated by the glucocorticoid receptor. **a** Viability courses of MCF7 cells treated for 24, 48, and 72 h with (**a**) ETOH, TNF, CORT, CORT + TNF, 10 μM RU486 (RU486), RU486 + TNF, RU486 + CORT, or RU486 + CORT + TNF; *p* < 0.005. **b** Same as in a) except with DEX instead of CORT. The values of all treatment groups were lower than that of the ETOH group; *P* < 0.005. **c** and **d** Luciferase (Luc) activity as an indirect measure of GR-dependent transcription. **c** Treatment with 1 μM CORT, 1 μM RU486 and 1 ng/mL TNF for 24 h and analyzed by Luc/β-Gal; *p* < 0.05. **d** Treatment with 1 μM DEX instead of CORT; *p* < 0.005. Data are presented as the mean ± standard deviation (SD); *n* = 3

To analyze whether the protective effect conferred by CORT or DEX is mediated by the binding of the hormone to its receptor, cell viability was evaluated in the presence of the GC antagonist RU486 at 24, 48, and 72 h. Using WB analysis, we verified that the cells expressed GR, estrogen receptor (ERα), and progesterone receptor (PRβ) and that they did not express human epidermal growth factor (HER2), as previously reported for this cell line (Additional file 4) [19, 20]. We observed that in the presence of the antagonist RU486, neither CORT nor DEX inhibited the cytotoxic effect of TNF (Fig. 2a and b, respectively). At 48 h, 50% of the MCF7 cells treated with CORT + TNF were viable, compared with 20% with of cells treated with TNF alone. With CORT + TNF + RU486 treatment, cell viability decreased to 16% (Fig. 2a). Similarly, 50% of the cells treated with DEX + TNF were viable, compared with 19% of cells treated with TNF alone. With DEX + TNF + RU486 treatment, viability decreased to 21% (Fig. 2b). These results suggest that the GR participates in the protective effect mediated by GCs.

Additionally, we wanted to determine whether the CORT- or DEX-induced GR transcription was affected by the TNF treatment. Thus, we measured luciferase activity as a reporter gene placed under the control of an artificial GR promoter by transfecting MCF7 cells with the pcDNA3.1-GR plasmid.

CORT resulted in a 4-fold increase in GR activation, while CORT + TNF resulted in a smaller, 2.8-fold, increase in GR activation (Fig. 2c). On the other hand, DEX promoted up to a 6-fold increase in GR activation, and the combination of DEX + TNF exerted a similar effect of a 5.7-fold increase (Fig. 2d). In contrast, in all treatment groups administered the antagonist, the increase in GR transcription ranged from 1.6-fold (for RU486 treatment) to 2.3-fold (for TNF + RU486 treatment). Taken together, these results indicated that, under experimental conditions in which GCs protect against TNF, the transcription of the GR is not affected. Thus, the GR could participate in the GC-mediated protection.

### The presence of cortisol and dexamethasone inhibits PARP1 processing in TNF-treated MCF7 cells and suggests the expression of inhibitor of apoptosis proteins

**Fig. 3:**
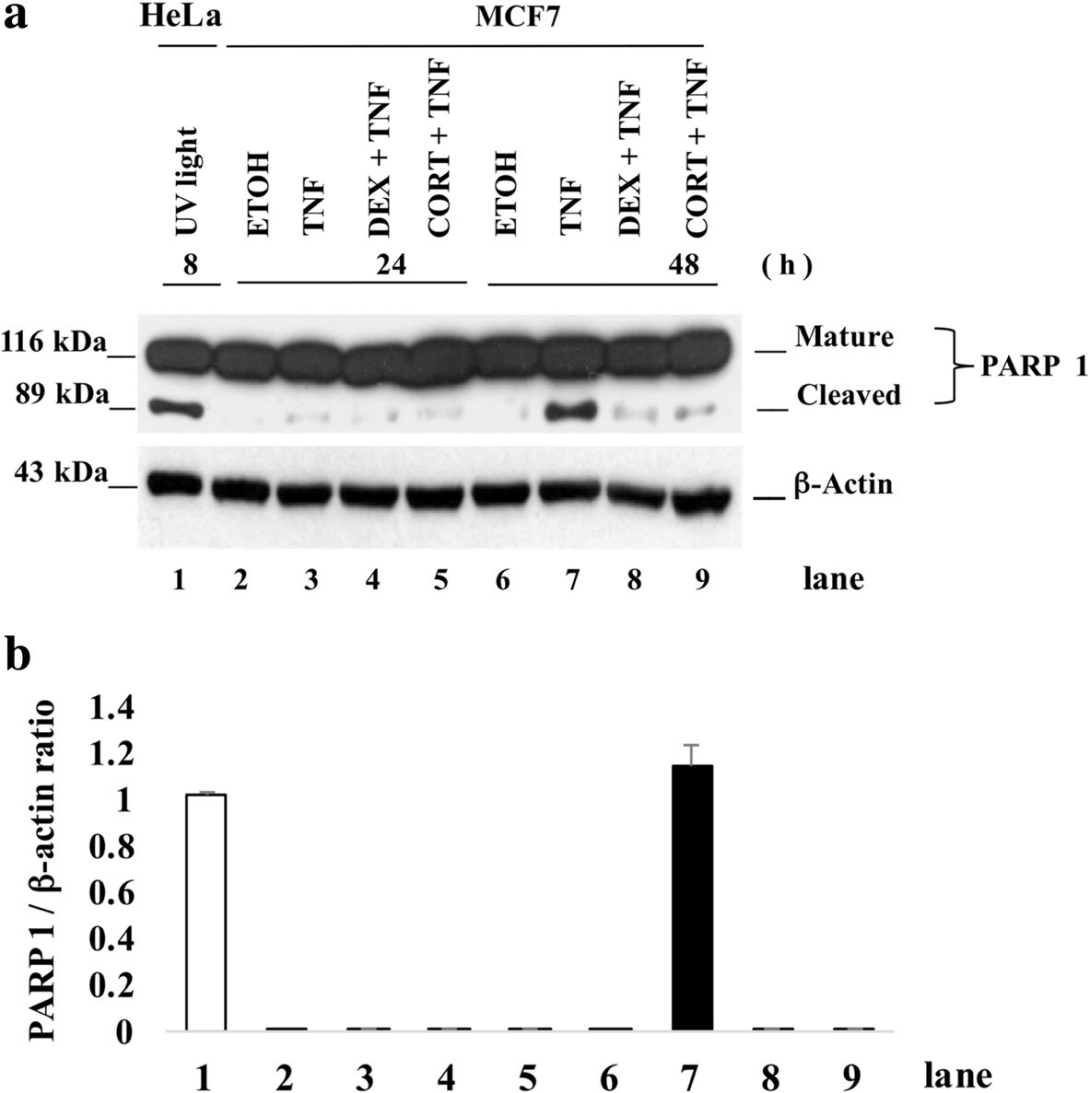
Death of TNF-treated MCF7 cells correlates with the processing of Poly (ADP-Ribose) Polymerase-1 (PARP1). **a** Western blot immunoassay of 30 μg of total proteins from MCF7 cells treated with ETOH, TNF, CORT + TNF or DEX + TNF for 24 h or 48 h (lanes 2–9). In lane 1, ultraviolet light (UV)-treated HeLa cells were used as a positive control for PARP1 processing; mature PARP1 (116 kDa) and cleaved PARP1 (89 kDa). **b** The cleaved PARP1/β-actin ratios of UV light-treated HeLa cells (lane 1) and of MCF7 cells treated with TNF for 48 h (lane 7) are shown. Data were normalized to the HeLa cell death model and are presented as the mean ± standard deviation (SD); *n* = 3

To establish whether the cytotoxic TNF effect resulted in cell death, we evaluated the cleavage of the enzyme PARP1, a marker of cell damage. We induced the apoptosis of HeLa cells by ultraviolet (UV) light as a positive control. The WB immunoassay (Fig. 3a) showed that in cultures treated with TNF, processed PARP1 was found at only 48 h (Fig. 3a, lane 7). Nevertheless, when TNF was combined with either CORT or DEX, there was no detectable cleavage of PARP1, suggesting a lack of enzymatic activity. Taken together, these results suggest that GCs interfere with the processing of PARP1.

### Cortisol and dexamethasone induced an increase in IAP mRNA levels in MCF7 cells treated with TNF

**Fig. 4:**
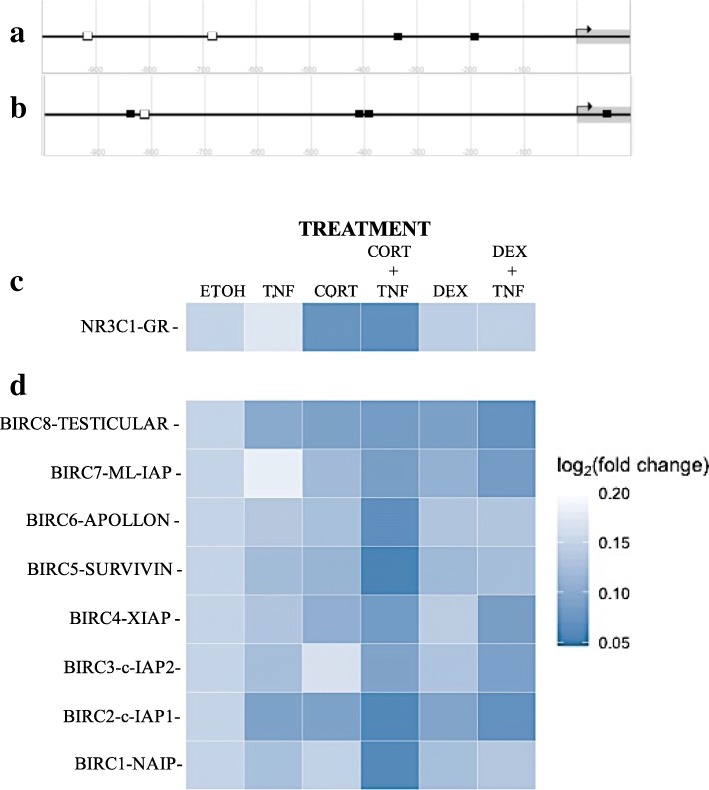
Diagrams of (**a**) the gene promoters of *c-IAP1* and (**b**) *XIAP* and heat maps of the log_2_ (fold change) mRNA levels of the *GR* and the *IAP* gene family. The gene promoters of a) *c-IAP1*, (**b**) *XIAP* and glucocorticoid receptor (*N3RC1*) response elements (GRE) are shown as white squares (*GRE* sequence: *AGAACANNNTGTTCT)*. NF-κB response elements (*ΚBRE* sequence: *AGTTGAGGGGACTTTCCCAGG*) of the p65 subunit are shown as black squares. The diagrams were modified from those available in the eukaryotic promoter database [51, 52]*.* **c** Glucocorticoid receptor. **d** The eight members of the mammalian *IAP* family. The data were normalized to ethanol (ETOH)-treated cells. The data represent the fold increase over the control (ETOH ± standard deviation [SD]); *n* = three independent experiments

GC-mediated attenuation of PARP1 cleavage could be due to an up-regulation of IAPs. IAPs inhibit effector caspases that are responsible for PARP1 cleavage. The promoters of c-IAP1 and XIAP have glucocorticoid response elements (GRE), as depicted (Fig. 4a and b). To confirm whether the GC-mediated protection from death activated by TNF-mediated cytotoxicity was associated with the increased expression of IAPs, we analyzed the mRNA content of all members of the mammalian IAP family and of the GR by qRT-PCR. Figure 4c and d show heat maps of the fold increase in mRNA content 18 h after the single or combined treatments (ETOH, TNF, CORT, DEX, or CORT + TNF, DEX + TNF) as determined by qRT-PCR analysis. The data were normalized to the ETOH group. The mRNA expression levels of all IAPs increased in the presence of both GCs. However, the most significant increase was observed in the combined treatment of CORT + TNF, which resulted in the following relative [10^3^] order of fold increase: Survivin (539 ± 0.1); c-IAP1 (176 ± 25); NAIP (102 ± 17); Apollon (26 ± 3); XIAP (4 ± 0.6); Ts-IAP (4 ± 0.2); ML-IAP (3 *±* 0.4), and c-IAP2 (1 ± 0.1). These results correspond with the effect of GCs; high levels of IAPs, especially c-IAP1 and XIAP, were maintained during protection. We analyzed the mRNA content of the GR with qRT-PCR (Fig. 4c). The CORT + TNF treatment induced a significant [10^3^]-fold increase (24 *±* 4) in GR mRNA, and treatment with CORT alone had a similar effect. In contrast, DEX alone or in combination with TNF had no effect. Interestingly, TNF alone led to the downregulation of GR transcripts. The combination of DEX + TNF induced a similar expression profile as TNF alone, except for APOLLON, SURVIVIN and NAIP, which displayed significantly decreased expression in the combination treatment. The expression profiles induced by treatment with CORT or DEX alone were similar; the expression of TESTICULAR-IAP, ML-IAP, APOLLON, SURVIVIN and c-IAP1 was increased. However, CORT resulted in the increased expression of XIAP, while DEX had no effect on XIAP. In contrast, CORT promoted the downregulation of c-IAP2, and DEX induced a mild increase in the expression of c-IAP2. Finally, TNF led to a mild increase in the expression of all IAPs, except for ML-IAP, which was downregulated.

### The protective effect of cortisol and dexamethasone maintains the intracellular levels of c-IAP1 and XIAP in TNF-treated MCF7 cells

**Fig. 5:**
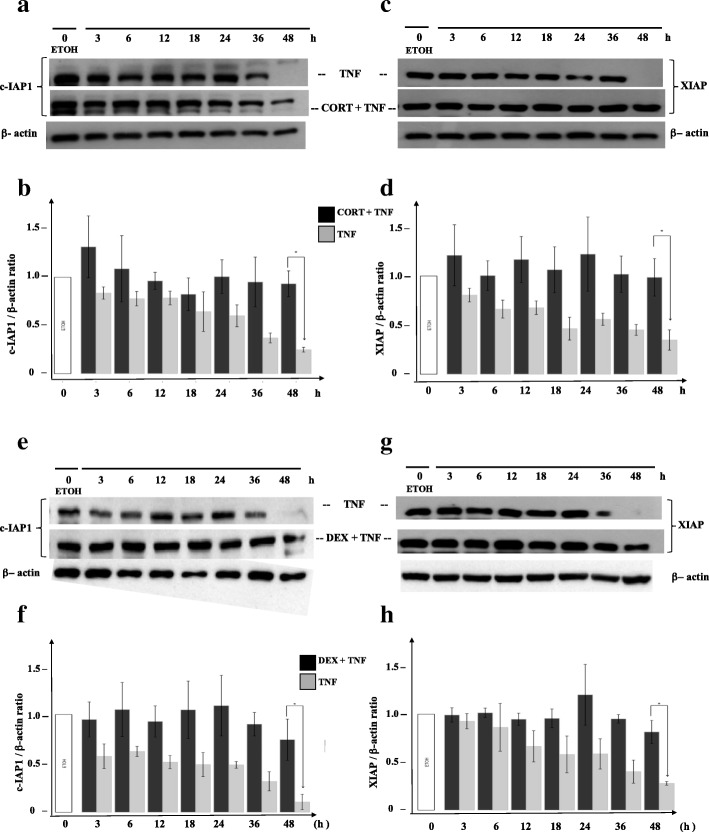
Cortisol and dexamethasone mediate sustained protein levels of c-IAP1 and XIAP during protection from TNF. Western blot immunoassay detecting c-IAP1 **a** or XIAP **c** using 30 μg of total proteins from MCF7 cells **a** and **c** treated with ethanol (ETOH), cortisol (CORT), or CORT + TNF for different lengths of time (3, 6, 12, 18, 24, 36, and 48 h). Lane 1 corresponds to cells treated with only vehicle (ETOH) for the indicated time **b** and **d**. Histograms representing panel **a** and panel **c** with the ratios of normalized c-IAP1 (panel **a**) and XIAP (panel **c**) levels to the β-actin signal (relative density [RD]) in cells treated with CORT. Vertical lines indicate the standard deviation (SD) of the mean (*p < 0.005). Data are presented as the mean ± SD; *n* = 3. Panels **e** and **g** are the same as **a** and **c** but with DEX instead of CORT. Panels **f** and **g** are the same as **b** and **d**, but with DEX instead of CORT. (*p < 0.005). Data are presented as the mean ± SD; *n* = 3

We wanted to know whether the lack of PARP1 cleavage under the GC + TNF combined treatment was due to the presence of IAPs. After evaluating the protein levels at different times from 3 to 48 h by WB analysis, we observed that, compared with the controls, the cells treated with TNF had decreased c-IAP1 levels (Fig. 5a, upper row; Fig. 5b, gray bars) and XIAP levels (Fig. 5c, upper row; Fig. 5d, gray bars). In contrast, the combined treatment of CORT + TNF led to the sustained expression of c-IAP1 (Fig. 5a, middle row; Fig. 5b, black bars) and XIAP (Fig. 5c, middle row; Fig. 5b, black bars). Similar results were obtained when cells were treated with TNF and with the combined treatment DEX + TNF (Fig. 5e and f for c-IAP1; Fig. 5g and h for XIAP).

In all cases, the combined GC + TNF treatment resulted in a significant difference in the c-IAP1 and XIAP contents. The protein content of both IAPs after 48 h with the combined treatment was not significantly different from to the protein content at 3 h; in all cases, the IAP content at 48 h was higher in cells with the combined treatment than in cells treated with only TNF (*p < 0.01). We also compared the protein content at 48 h in the control vs ETOH groups (Additional file 5). These results suggest that GCs either prevent IAP degradation or promote IAP synthesis and could be related to the protection against TNF-mediated cytotoxicity.

### siRNAs against *c-IAP1* and *XIAP* inhibited the protection conferred by cortisol or dexamethasone

**Fig. 6:**
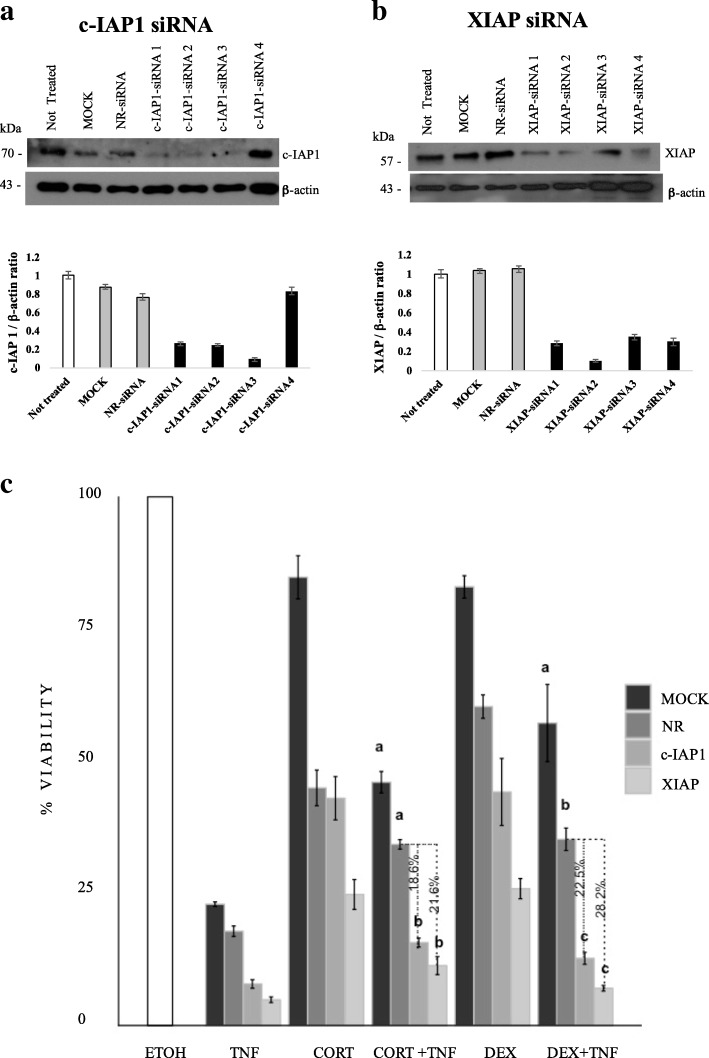
GC-mediated protection against TNF is diminished by inhibiting c-IAP1 or XIAP expression. Western blot immunoassay of MCF7 cells transfected with the four siRNAs against c-IAP1 (**a**) or XIAP (**b**) for 18 h. MOCK: no siRNA; NR-siRNA: nonrelated siRNA; c-IAP1-siRNA and XIAP-siRNA, from 1 through 4 (with four different sequences each). The histograms present the ratios of normalized siRNA-targeted c-IAP1 and XIAP expression to normalized β-actin expression. For transfections, we used the lowest IAP/β-actin ratio. Vertical lines indicate the standard deviation of the mean (*p < 0.005). Data are presented as the mean ± standard deviation [SD]; *n* = 3. **c**) Viability assay of MCF7 cells treated for 24 h with the indicated treatments. An average of two independent experiments was performed with each sample conducted in sextuplicate. Vertical lines represent the standard deviation of the mean; *p < 0.05. Letters indicate distinct groups based on the post hoc statistical comparison (*p* < 0.05). If groups share at least one letter, they do not statistically differ. Data are presented as the mean ± SD; *n* = 2

To verify whether the sustained levels of c-IAP1 and XIAP contribute to the protection conferred by CORT or DEX in TNF-treated MCF7 cells, we interfered with their expression by administering four siRNAs against both the *c-IAP1* and *XIAP* (QIAGEN) genes: c-IAP1-siRNA 1, c-IAP1-siRNA 2, c-IAP1-siRNA 3, and c-IAP1-siRNA 4 (Fig. 6a); XIAP-siRNA 1, XIAP-siRNA 2, XIAP-siRNA 3, and XIAP-siRNA 4 (Fig. 6b). We selected c-IAP1-siRNA 3 and XIAP-siRNA 2 for cell transfection, based on their ability to diminishing protein content as observed by the IAP/β-actin ratio in the WB histogram (Fig. 6a and b). Subsequently, we transfected MCF7 cells as described in the experimental procedures with the chosen siRNAs. Then, 24 h post-transfection, the cells were treated with ETOH, TNF, CORT + TNF or DEX + TNF to evaluate the importance of *IAP* expression in the protection conferred by GCs. Cell viability was evaluated 18 h post-treatment. Interference with *IAP* expression significantly decreased (*p < 0.05) the anti-cytotoxic effects of CORT and DEX (Fig. 6c and Additional file 6). The protection conferred by CORT + TNF decreased by 18.6% with c-IAP1 knockdown and by 21.6% with XIAP knockdown, while the protection conferred by DEX + TNF decreased by 22.5% with c-IAP1 knockdown and by 28.2% with XIAP knockdown (Additional file 6).

With both GCs, these decreases were statistically significant (*p* < 0.05) and demonstrated that the presence of these two IAPs is important for anti-cytotoxic protection.

## Discussion

GCs are steroid hormones responsible for modulating basic metabolism, cell survival, and homeostasis, as well as for regulating immune and neural functions [21]. CORT is the best known adrenal steroid and is more potent than corticosterone, while synthetic agonists such as DEX are even more potent than CORT [22]. CORT biosynthesis occurs in the fasciculate layer of the adrenal gland; it has a short half-life of approximately 90 min, and the expression of CORT has a circadian rhythm [23, 24]. The effect of GCs on vital functions is frequently used in clinical settings to strengthen weak or terminal patients. All of these effects are mediated by the transcriptional activation of GR target genes [25–27].

**Fig. 7:**
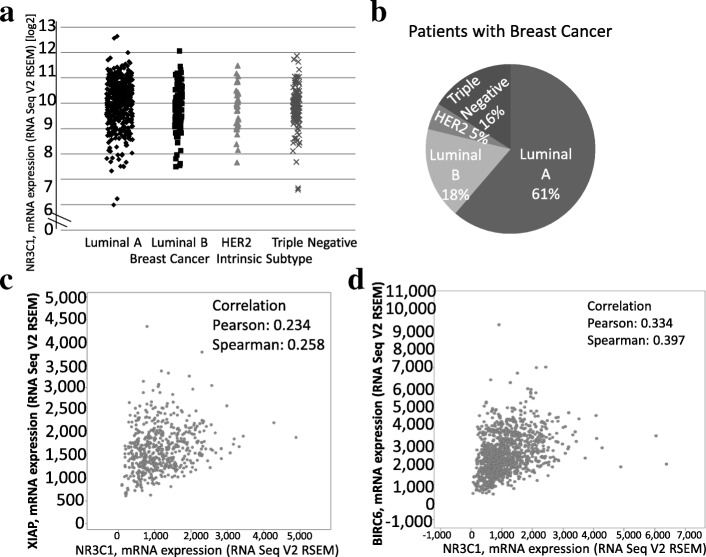
*NR3C1* mRNA expression and coexpression with other genes in samples from breast cancer patients. **a** *NR3C1* mRNA expression in 721 samples from patients with different intrinsic subtypes of breast cancer. The ranges of *ESR1* and *AR* expression are included for comparison and indicated by brackets. **b** Percentage of patients expressing each intrinsic subtype of breast cancer in panel **a**. **c** and **d** Coexpression of *NR3C1* mRNA with *XIAP* or *BIRC6* mRNA in the data of 1100 tumor samples from breast cancer patients

In contrast, the role of GCs in cancer is currently being established. IHC experiments have demonstrated that the GR localized in the nucleus of tumor biopsies from different tissues of origin [28]. Regarding breast cancer, the mRNA of the GR gene (*NR3C1*) is abundantly present in patient tumor biopsies of all intrinsic subtypes (Fig. 7a). Although we did not examine GR protein expression levels, it has been reported that ER/PR-positive and low-grade tumors express high levels of GR [29]. Moreover, only 3% of the 721 patient samples in our analysis had a mutation in *NR3C1* (data not shown). This finding suggests that GR expression in tumoral cells and in nontransformed cells associated with tumors contributes to the biological function of breast cancer, and a detailed analysis of GC signaling could reveal genes that drive breast cancer and identify therapeutic targets [30]. Although the frequency of each subtype in the database consulted in this study overrepresents the luminal A subtype (61%) and underrepresents the HER2 subtype (5%), the database generally coincides with the reported population prevalence [31]. It is important to recognize that gene expression in tumor samples, such as those described in Fig. 7, does not imply that GR expression is restricted to tumor cells; the GR could be expressed in nontransformed tumor-associated cell types (endothelial cells, fibroblasts, or immune cells). In addition, GR expression has been reported in nontransformed cells associated with luminal A cells in human breast cancer, such as cancer-associated fibroblasts (CAFs) positive for smooth muscle actin (SMA), which present a myofibroblast (PMY) phenotype. The expression of GR in breast CAFs could be an interesting target for future therapy to regulate the tumoral breast microenvironment [32]. It should be noted that we used RNA-seq expression data rather than proteomic information and that the database was limited to 77 patients. The protein product of *NR3C1* possesses a biological function. It is possible that transcriptomic and proteomic data do not correlate and that our conclusions may not be valid.

Our in vitro results indicate that the combined treatment of CORT + TNF induces the expression of all members of the IAP family (Survivin>c-IAP1 > NAIP>Apollon>XIAP >ML-IAP > Ts-IAP > c-IAP2), and analysis of the TCGA database reveals a different order of correlation between GR and IAP family members, except for Survivin, which exhibits a negative correlation (Apollon, XIAP, NAIP, c-IAP1 > ML-IAP, Ts-IAP) (Fig. 7c and d; Table 1). It is interesting that we identified a positive correlation between GR and IAP family members both in vitro and in vivo in the RNA-seq data; this result could be related to the cytoprotective effect of the IAP family. While it is clear in vitro that IAP expression is dependent on simultaneous stimulation with CORT + TNF, in vivo we can refer to only the positive correlation of the expression of the GR and the IAP family. The discrepancies in the relative order of the expression of the GR and IAP family members in vitro and in vivo could be due to the complexity of in vivo signals (hormones, metabolic changes, and tumor microenvironment). However, the coexpression of *GR* and *IAP* genes may not indicate the direct and exclusive induction of the latter by the former. Other transcription factors could be required for IAP induction. It has yet to be determined whether the GR binds to the promoters of IAP family members. Even if a direct interaction does exist, the level that this modulation of gene expression occurs has yet to be described (i.e., whether the GR modulates the transcription, translation, mRNA half-life or protein degradation of IAPs).

**Table 1:** Pearson correlation coefficient of the coexpression of *NR3C1* vs *IAP* family genes

Gene coexpressed with *NR3C1*	Pearson correlation (*R*)
*Apollon*	0.344
*XIAP*	0.234
*BIRC3 (c-IAP2)*	0.210
*NAIP*	0.210
*BIRC2 (c-IAP1)*	0.141
*BIRC7 (ML-IAP)*	0.025
*BIRC8 (Ts-IAP)*	−0.002
*BIRC5 (Survivin)*	− 0.249

The data used to produce this table were obtained from tumor samples of breast cancer patients and were procured from the Breast Invasive Carcinoma (TCGA, Provisional) database in cBioportal. In our in vitro experiments, we found that TNF-mediated cytotoxicity is inhibited by GCs (CORT or DEX) in MCF7 breast cancer cells. This protective effect is dependent on the expression of IAP family members (c-IAP1 and XIAP). A key issue arising from these results is whether GCs induce an increase in cell proliferation, inhibit cell death, or both to counteract TNF cytotoxicity. GCs have been reported to be able to arrest the cell proliferation of MCF7 cells [33]. In our results, GC treatments alone reduced cell proliferation (Additional file 2). When GCs were combined with TNF, cell proliferation rates increased by 27 and 32% for CORT and DEX, respectively (Fig. 1a). These improvements in the cell proliferation rate with the combined treatments suggests that the decrease in cell proliferation induced by GCs is not directly involved in the protective effect. We postulate that additional mechanisms related to the transcriptional activity of GCs could be related to cell survival, such as anti-apoptotic mediators; in fact, GCs have been reported to induce IAP expression [34]. This dual effect has also been reported in other studies, and the effect on tumors has been controversial; GCs have been described as both promoters and inhibitors of tumor proliferation [35]. Some clinical studies have reported that GCs can suppress tumor progression and metastasis, especially in endocrine responsive tumors [36]. In contrast, other clinical studies reported that GCs in ER-negative tumors are associated with chemo-resistance and worsened prognosis [37]. This controversial effect might be a result of various factors, including different levels of GR expression, which could be used as a prognostic biomarker in lower grade, ER-positive luminal breast cancer. Moreover, GCs effect could be influenced by tumor size, stage and grade [3], GC doses, response to endocrine therapy and inflammatory signaling, such as an interaction with TNF as shown in our results. These differences are of clinical relevance since synthetic GCs, particularly DEX, are routinely included in chemotherapy therapies for breast cancer and other nonhematologic malignancies to reduce side effects, such as nausea, anorexia and hypersensitivity [6]. However, RU486 is not used in the clinic for breast cancer treatment, and it was used in this study for its inhibitory effect on GR [25].

We used RU486, a partial antagonist of GR, to probe whether GC-mediated protection requires interaction with the GR (Fig. 2a and b). The fact that the GC-mediated protection against TNF treatment was diminished in the presence of RU486 suggests that the GR is required for this protection. The use of RU486 as a specific antagonist is limited by the fact that when used alone, it can act as a partial agonist (Fig. 2c and d) [38]. Therefore, to provide further support for the involvement of the GR-IAP pathway, we utilized siRNA against c-IAP1 and XIAP (Fig. 6).

There are several molecular markers to detect caspase-dependent cell death. PARP1 is a direct substrate of the effector caspases 3, 6 & 7; thus, PARP1 cleavage is a good indicator of caspase proteolytic activity. Phosphatidyl serine translocation from the cytoplasmic lamella to the extracellular membrane lamella is also a frequently used marker of apoptosis, but it is not directly linked to caspase activation. Caspase activation leads to DNA fragmentation and inhibits DNA repair pathways such as the pathway that requires PARP1 [39]. In MCF7 cells, TNF treatment has been documented to promote the processing of PARP1 [16]. A key observation included the modest processing of PARP1 in cells treated with GCs + TNF (Fig. 3a, lanes 8 and 9) compared with cells treated with only TNF (Fig. 3a, lane 7). These results could imply that the proteases involved in PARP1 cleavage were inhibited, most likely by IAPs.

Apoptosis is a programmed cell death mechanism that requires the activation of a family of cysteine proteases [40]. In MCF7 cells, TNF induces the activation of these proteases, leading to the processing of the 116 kDa PARP1 (poly [ADP-ribose] polymerase-1) into the 89 kDa form. The MCF7 cell line is the only breast cancer cell line reported to have a cytotoxic response to TNF [14–16]. We suggested the involvement of IAPs and determined whether this protection is associated with the inhibition of PARP1 processing.

It is interesting to note that GCs alone induced an increase in the mRNA levels of some IAPs (Testicular-IAP, ML-IAP, Survivin and c-IAP1). TNF alone had a selective effect, inducing only Testicular-IAP and c-IAP1 and exerting a mild effect on other IAPs. However, the combination of DEX + TNF appears to have an additive effect. In contrast, the combination of CORT + TNF appears to have a synergistic effect on IAP expression levels, especially for Survivin and NAIP.

The relative increase in the expression of XIAP and c-IAP1 was also evaluated by WB analysis. However, the protein level of these IAPs was not as exacerbated as their mRNA levels. A possible explanation for this could be post-transcriptional regulatory effects such as mRNA stability, selection of mRNA transcripts for translation and high turn-over of the proteins after translation. Interestingly Nestal de Moraes et al., showed that doxorubicin treatment in MCF7 cells resulted in poorly correlated effects on the mRNA and protein levels of Survivin and XIAP [41]. Moreover, Chunsen Xu et al., reported that the mRNA and protein levels of survivin do no correlate in breast cancer tumor tissues, but the mRNA levels are a predictor of poor prognosis in patients with breast cancer [42].

TNF treatment induced a decrease in IAP protein levels (Fig. 5a, c, e and g, upper row), and cells treated with GCs + TNF maintained their IAP protein levels (Fig. 5a, c, e and g, middle row). This effect could result from interference with IAP degradation, increased IAP transcription, or a combination of both. IAPs act as competitive inhibitors of apoptosis, resulting in the delay or the complete prevention of cell death. IAPs interact with caspases through the baculovirus inhibitor of apoptosis protein repeat domain (BIR) domain and control the assembly of the “ripoptosome”. Specifically, c-IAP1 and XIAP have three BIR domains that selectively interact with caspases to control cell death [43]. Homeostasis is regulated by maintaining the equilibrium between the synthesis and degradation of proteins and is mediated by poly-ubiquitination and proteasome degradation [39]. We measured the processing of PARP1 substrate (Fig. 3, lane 7). The addition of CORT or DEX to TNF treatment prevented PARP1 cleavage (Fig. 3, lanes 8 and 9). We postulated that this interference could be explained if GCs induced the expression of IAPs [34]. The promoters of c-IAP1 (Fig. 4a) and XIAP (Fig. 4b) contain canonical GC response elements (GRE) (Fig. 4a and b, white squares), which could induce their expression in the presence of CORT and DEX.

Additionally, these promoters also contain NF-κB-response elements (ΚBRE) (Fig. 4, black squares). It is noteworthy that it was previously reported that TNF treatment activates NF-κB and that the protection provided by DEX against TNF is dependent on NF-κB activation in MCF7 cells [16]. Additionally, this protection was reported in mouse fibroblasts L929 cells [44].

The transient expression of siRNA targeting c-IAP1 and XIAP led to a significant decrease in protein content (Fig. 6a and b) and was sufficient to interfere with the protection conferred by CORT and DEX. It is interesting to note that interfering with XIAP resulted in a stronger effect than interfering with c-IAP1 (Fig. 6c). Considering that the GC-induced protection against TNF-mediated cytotoxicity was correlated with a sustained level of IAPs, it is likely that this protection is the result of a combined effect of at least these two IAPs. This result provides support for the original observation by Messmer [15] that DEX protects against TNF-mediated cytotoxicity while sustaining IAP proteins content. Similar results have been reported for DEX in A549 cells; DEX protected against TRAIL and anticancer drugs through mediating c-IAP2 expression [45]. In vivo, DEX has been reported to interfere with apoptosis in the central nervous system and in testicular germ cells [46]. It is likely that, under these in vitro and in vivo conditions, the expression of diverse forms of IAPs (c-IAP1 and XIAP) provides protection against apoptosis. However, whether the anti-apoptotic effect of GCs occurs in a more clinically relevant setting, such as cancer drug-mediated cytotoxicity, is an intense area of debate [7]. GCs are the most common antiemetics and are administered shortly before chemotherapy to counteract the secondary effects of chemotherapy [6]. In the case of MCF7 cells that represent luminal A breast cancer, in vitro evidence suggests that GCs inhibit the effect of paclitaxel [47]. Nevertheless, GCs alone have anticancer effects, such as inhibiting angiogenesis in vitro [48] and cell proliferation [49]; the latter effect was recently associated with the capacity of cells to re-establish the expression of clock genes such as *BMAL* [50], although this has yet to be proven in the case of breast cancer cells. Moreover, studies in humans that evaluated the effect of GCs on chemotherapy effectiveness in breast cancer patients, to the best of our knowledge, have not yet been carried out. The time of chemotherapy administration could also be relevant for its effectiveness, considering the circadian nature of the serum levels of CORT [23].

ER+ breast cancer represents more than 60% of all breast cancer patients; however, this clinical group is heterogeneous. Considering our findings and those of other studies that support a relevant role of the GR in this type of cancer, it would be useful to revisit the relevance of the expression profile of nuclear receptors (AR, MR, VDR, TDR, etc.), coactivators, corepressors and isoforms. This kind of analysis could lead to the identification of subgroups and the detection of differences in the prognosis and treatment response of these patients.

## Conclusions

**Fig. 8:**
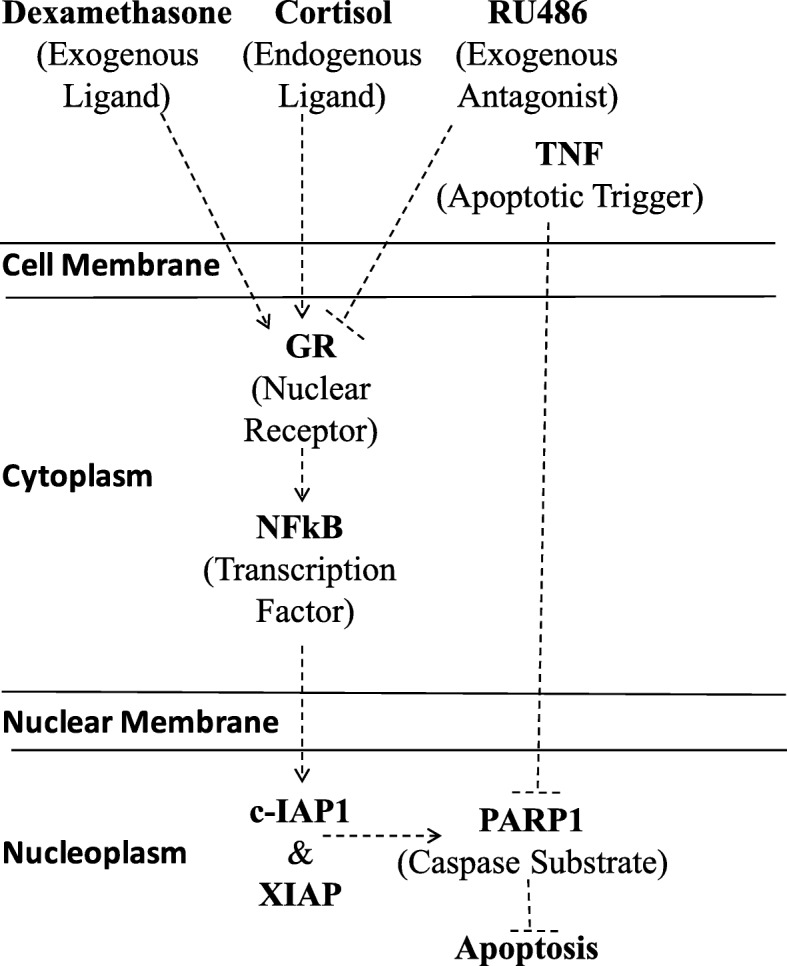
Mechanistic map of the effect of TNF and GCs on MCF7 cells. Glucocorticoid receptor (GR), mifepristone (RU486), poly-adenyl ribosyl polymerase-1 (PARP1), nuclear factor-kappa light chain in B cells (NF-κB), cellular 1 inhibitor apoptosis protein (c-IAP1), X chromosome-linked inhibitor apoptosis protein (XIAP), and tumor necrosis factor (TNF)

Hormone therapy in breast cancer is limited to the inhibition of estrogen signaling. Cumulative evidence indicates a role for endogenous and exogenous GCs in tumor progression. Here, we underscore the role of IAPs in the mechanism by which CORT and DEX interfere with the TNF-mediated cytotoxic effect on MCF7 breast cancer cells (Fig. 8). We also show that IAPs correlate with GR expression using public transcriptomic data from the tumors of patients. Our study should motivate further analysis on the administration of GCs or anti-GCs to breast cancer patients. Additionally, the gene expression levels of members of the *GR* and *IAP* families could be evaluated as prognostic markers for breast cancer therapy.

## Additional files

Additional file 1: Sequences of the oligonucleotide primers used in qRT-PCR. List of 22 oligonucleotides, primer sequences, position, melting temperature (Tm), and PCR product size used for qRT-PCR in the project. (DOCX 97 kb)

Additional file 2: The addition of glucocorticoids exerts changes in the proliferation of MCF7 cells. Micrograph of the confluent culture of MCF7 tumor cells treated for 48 h with 0.1% ethanol (ETOH), 10 μM cortisol (CORT), and 10 μM dexamethasone (DEX). Arrows show mitotic figures. Representative images were taken under X100 magnification. (DOCX 14283 kb)

Additional file 3: Dexamethasone and cortisol block the cytotoxic effect of TNF on MCF7 cells. Histogram of NCI at 24, 48, and 72 h, obtained from the XCELLigence system. Vertical bars represent the SD of each measurement, and asterisks represent significant differences between treatments (*p < 0.001). Data are presented as the mean ± SD. *n* = 3. (DOCX 279 kb)

Additional file 4: Western blot immunoassay for the detection of nuclear receptors in MCF7 and BT-474 breast cancer cells. Total protein extracts (30 μg) from MCF7 or BT-474 cells. Arrows show the expected molecular weight for each of the analyzed receptors (HER2/neu, human epidermal growth factor receptor type 2; GR, glucocorticoid receptor; PR, progesterone receptor; ER, estrogen receptor). (DOCX 498 kb)

Additional file 5: Cortisol and dexamethasone mediate sustained protein levels of c-IAP1 and XIAP during protection against TNF. The control and vehicle (ETOH) panel from Fig. 5a, c, e and g at 0 h and 48 h of ETOH, GCs and TNF treatment. (DOCX 431 kb)

Additional file 6 siRNA against c-IAP1 or XIAP decreased the cytotoxic protection of GCs. Values represent the % viability at 18 h post-treatment. MOCK: Transfection without siRNA. NR-siRNA: Transfection with nonrelated siRNA. c-IAP1-siRNA: Transitory transfection with siRNA targeting c-IAP1 transcripts. XIAP-siRNA: Transitory transfection with siRNA targeting c-XIAP transcripts. The siRNAs selected for this experiment resulted in the lowest expression of their target IAP (as shown in Fig. 6) (*): Represents the % loss of protection in cells with siRNA-mediated inhibited c-IAP1 and XIAP vs cells treated with CORT + TNF + NR-siRNA or DEX + TNF + NR-siRNA. (DOCX 130 kb)

## Abbreviations

BIR: Baculovirus inhibitor of apoptosis protein repeat domain
c-IAP1: cellular inhibitor of apoptosis protein 1
CORT: Cortisol
DEX: Dexamethasone
DUSP1: Dual specificity phosphatase 1
ELISA: Enzyme-linked immunosorbent assay
ERɑ: Estrogen receptor alpha
ETOH: Ethanol
FAS: First apoptosis signal receptor
FBS: Fetal bovine serum
GCs: Glucocorticoids
GR: Glucocorticoid receptor alpha
GRE: Glucocorticoid response elements
HER2: Human epidermal growth factor receptor 2
IAP: Inhibitor of apoptosis proteins
IHC: Immunohistochemistry
MFC7: Michigan cancer foundation 7 cell line
NCI: Normalized cell index
NF-κB: Nuclear factor-kappa B
*NR3C1*: Glucocorticoid receptor gene
PARP1: Poly (ADP-ribose) polymerase-1
PRβ: Progesterone receptor beta
RNAi: RNA interference
RPMI: Roswell Park Memorial Institute
RU486: Mifepristone
SGK1: Serum/glucocorticoid regulated kinase 1
siRNA: Short interfering RNA
TCGA: The Cancer Genome Atlas
TNBC: Triple negative breast cancer
TNF: Tumor necrosis factor
XIAP: X-linked inhibitor of apoptosis protein
