# Supplementary material for: Glucocorticoid-dependent expression of IAP participates in the protection against TNF-mediated cytotoxicity in MCF7 cells
Source: BMC Cancer. 2019 Apr 15;19:356. doi: 10.1186/s12885-019-5563-y (PMC6466787; doi:10.1186/s12885-019-5563-y)
Supplement: Supplementary file 1 — Sequences of the oligonucleotide primers used in qRT-PCR. List of 22 oligonucleotides, primer sequences, position, melting temperature (Tm), and PCR product size used for qRT-PCR in the project. (DOCX 97 kb) [file 12885_2019_5563_MOESM1_ESM.docx]

| N° | Oligonucleotide | Sequence | Tm (°C) | PCR product size | Position |
| --- | --- | --- | --- | --- | --- |
| 1 | *NAIP F* | 5´-TTC TTG CCC TGA AAA CTG CT-3´ | 59.99 | 169 | 3312-3480 |
| 2 | *NAIP R* | 5´-CGT ATT GGG AAG TGG ATG CT-3´ | 59.96 |  |  |
| 3 | *c-IAP1 F* | 5´-TGA ACG AAA AAG AGG TAG CAC-3´ | 59.99 | 294 | 3454-3507 |
| 4 | *c-IAP1 R* | 5´-CCG GTA TTA GTA CAA TGC CAA A-3´ | 59.99 |  |  |
| 5 | *c-IAP2 F* | 5´-CCA AGT GGT TTC CAA GGT GT-3´ | 59.86 | 119 | 3733-3851 |
| 6 | *c-IAP2 R* | 5´-TGG GCT GTC TGA TGT GGA TA-3´ | 60.07 |  |  |
| 7 | *XIAP F* | 5´-GTG CGG TGC TTT AGT TGT CA-3´ | 59.91 | 294 | 340-633  exon 2 |
| 8 | *XIAP R* | 5´-AGG GTT CCT CGG GTA TAT GG-3´ | 60.03 |  |  |
| 9 | *Survivin F* | 5´-GGA CCA CCG CAT CTC TAC AT-3´ | 59.96 | 183 | 166-348 |
| 10 | *Survivin R* | 5´-TCC TCT ATG GGG TCG TCA TC-3´ | 59.89 |  |  |
| 11 | *Apollon F* | 5´-TGT CAC CTC TTC AGC CAC AG-3´ | 60.02 | 112 | 6571-6682 |
| 12 | *Apollon R* | 5´-GCC ACA TAA TCC AGC AAC CT-3´ | 59.96 |  |  |
| 13 | *ML-IAP F* | 5´-TGG CCT CCT TCT ATG ACT GG-3´ | 60.21 | 101 | 445-545 |
| 14 | *ML-IAP R* | 5´-GCA CCT CAC CTT GTC CTG AT-3´ | 60.12 |  |  |
| 15 | *Ts-IAP F* | 5´-TCA CTT GAG GGA GCT CTG GT-3´ | 59.99 | 274 | 1504-1777 |
| 16 | *Ts-IAP R* | 5´-CAG GGC TGA TTT CTC TCT GC-3´ | 60.46 |  |  |
| 17 | *GR F* | 5´-TAC CCT GCA TGT ACG ACC AA-3´ | 59.99 | 212 | 2399-2610 |
| 18 | *GR R* | 5´-TCC TTC CCT CTT GAC AAT GG-3´ | 60.04 |  |  |
| 19 | *TBP F* | 5´-CCA CAG CTC TTC CAC TCA CA-3´ | 59.99 | 254 | 493-2826 |
| 20 | *TBP R* | 5´-CTC ATG ATT ACC GCA GCA AA-3´ | 59.99 |  |  |
| 21 | *GUS B F* | 5´-AAA CGA TTG CAG GGT TTC AC-3´ | 59.99 | 171 | 399-610 |
| 22 | *GUS B R* | 5´-CTC TCG TCG GTG ACT GTT CA-3´ | 59.99 |  |  |

**Additional file 1.**
