# Supplementary material for: Glucocorticoid-dependent expression of IAP participates in the protection against TNF-mediated cytotoxicity in MCF7 cells
Source: BMC Cancer. 2019 Apr 15;19:356. doi: 10.1186/s12885-019-5563-y (PMC6466787; doi:10.1186/s12885-019-5563-y)
Supplement: Supplementary file 2 — The addition of glucocorticoids exerts changes in the proliferation of MCF7 cells. Micrograph of the confluent culture of MCF7 tumor cells treated for 48 h with 0.1% ethanol (ETOH), 10 μM cortisol (CORT), and 10 μM dexamethasone (DEX). Arrows show mitotic figures. Representative images were taken under X100 magnification. (DOCX 14283 kb) [file 12885_2019_5563_MOESM2_ESM.docx]

**Additional file 2.**
