# Supplementary material for: Glucocorticoid-dependent expression of IAP participates in the protection against TNF-mediated cytotoxicity in MCF7 cells
Source: BMC Cancer. 2019 Apr 15;19:356. doi: 10.1186/s12885-019-5563-y (PMC6466787; doi:10.1186/s12885-019-5563-y)
Supplement: Supplementary file 3 — Dexamethasone and cortisol block the cytotoxic effect of TNF on MCF7 cells. Histogram of NCI at 24, 48, and 72 h, obtained from the XCELLigence system. Vertical bars represent the SD of each measurement, and asterisks represent significant differences between treatments (*p < 0.001). Data are presented as the mean ± SD. n = 3. (DOCX 279 kb) [file 12885_2019_5563_MOESM3_ESM.docx]

**Additional file 3.**
