# Supplementary material for: Glucocorticoid-dependent expression of IAP participates in the protection against TNF-mediated cytotoxicity in MCF7 cells
Source: BMC Cancer. 2019 Apr 15;19:356. doi: 10.1186/s12885-019-5563-y (PMC6466787; doi:10.1186/s12885-019-5563-y)
Supplement: Supplementary file 4 — Western blot immunoassay for the detection of nuclear receptors in MCF7 and BT-474 breast cancer cells. Total protein extracts (30 μg) from MCF7 or BT-474 cells. Arrows show the expected molecular weight for each of the analyzed receptors (HER2/neu, human epidermal growth factor receptor type 2; GR, glucocorticoid receptor; PR, progesterone receptor; ER, estrogen receptor). (DOCX 498 kb) [file 12885_2019_5563_MOESM4_ESM.docx]

**Additional file 4.**
