# Supplementary material for: Glucocorticoid-dependent expression of IAP participates in the protection against TNF-mediated cytotoxicity in MCF7 cells
Source: BMC Cancer. 2019 Apr 15;19:356. doi: 10.1186/s12885-019-5563-y (PMC6466787; doi:10.1186/s12885-019-5563-y)
Supplement: Supplementary file 5 — Cortisol and dexamethasone mediate sustained protein levels of c-IAP1 and XIAP during protection against TNF. The control and vehicle (ETOH) panel from Fig. 5a, c, e and g at 0 h and 48 h of ETOH, GCs and TNF treatment. (DOCX 431 kb) [file 12885_2019_5563_MOESM5_ESM.docx]

**Additional file 5.**
