# Supplementary material for: Glucocorticoid-dependent expression of IAP participates in the protection against TNF-mediated cytotoxicity in MCF7 cells
Source: BMC Cancer. 2019 Apr 15;19:356. doi: 10.1186/s12885-019-5563-y (PMC6466787; doi:10.1186/s12885-019-5563-y)
Supplement: Supplementary file 6 — siRNA against c-IAP1 or XIAP decreased the cytotoxic protection of GCs. Values represent the % viability at 18 h post-treatment. MOCK: Transfection without siRNA. NR-siRNA: Transfection with nonrelated siRNA. c-IAP1-siRNA: Transitory transfection with siRNA targeting c-IAP1 transcripts. XIAP-siRNA: Transitory transfection with siRNA targeting c-XIAP transcripts. The siRNAs selected for this experiment resulted in the lowest expression of their target IAP (as shown in Fig. 6) (*): Represents the % loss of protection in cells with siRNA-mediated inhibited c-IAP1 and XIAP vs cells treated with CORT + TNF + NR-siRNA or DEX + TNF + NR-siRNA. (DOCX 130 kb) [file 12885_2019_5563_MOESM6_ESM.docx]

**Additional file 6.**
